# Posterior Nutcracker Syndrome with Left Renal Vein Duplication: A Rare Cause of Haematuria in a 12-Year-Old Boy

**DOI:** 10.1155/2012/849681

**Published:** 2012-08-02

**Authors:** J. Preza Fernandes, Rui Amorim, M. J. Gomes, V. Oliveira, A. Reis, J. Ribeiro-Castro

**Affiliations:** ^1^Serviço de Urologia, Hospital de Santo António, Centro Hospitalar do Porto, 4100 Porto, Portugal; ^2^Serviço de Urologia, Centro Hospitalar Vila Nova de Gaia Espinho, 4434-502 Vila Nova de Gaia, Portugal; ^3^Serviço de Cirurgia Pediátrica, Centro Hospitalar do Porto, 4050-111 Porto, Portugal

## Abstract

The nutcracker syndrome (NCS) is a rare cause of haematuria. It embraces an extended nonpathognomonic spectrum of symptoms that imply a difficult diagnosis. Ultimately it may be associated with substantial morbidity and even life-threatening events. We report a rare cause if a 12-year-old boy who presented with a history of frequent intermittent episodes of painless constant haematuria. The cystoscopy showed a bloody urine ejaculate from the left ureter meatus. The Doppler ultrasonography showed turbulent pattern of venous blood flow of the posterior renal vein branch behind the aorta. The abdominopelvic computer tomography (apCT) revealed left renal vein (LRV) duplication with a dilated retroaortic branch, entrapped between the aorta and the vertebral column, promoting the renal nutcracker syndrome. The patient was initially hospitalized and managed with oral iron supplements and continuous saline bladder irrigation, not requiring additional treatment. The child is currently asymptomatic, with haemoglobin value returning to normal and therefore proposed to conservative management with close followup. The authors present a case report of episodic haematuria caused by a rare entity—posterior nutcracker syndrome with renal vein duplication.

## 1. Introduction

Nutcracker syndrome is a rare cause of haematuria caused by the left renal vein (LRV) entrapment [1–5], most usually between the aorta and the superior mesenteric artery (SMA), known as anterior nutcracker syndrome. Sometimes a retroaortic position of the LRV also promotes an entrapment, this time between the aorta and the vertebral column, which is named posterior nutcracker syndrome [6]. Although the first clinical report was made by El-Sadr and Mina [1] in 1950, the term *nutcracker* is credited to de Schepper in 1972 [2] but was first used by Chait et al. in 1971 [3].

This term must be distinguished from the Nutcracker Phenomenon [4]. This is a relatively common anatomical variance, in which the patient stays asymptomatic, and it is often diagnosed in an occasional imaging exam. It usually affects women more than men [1] and in most cases present in the 3rd or 4th decades of life.

When symptomatic this syndrome is manifested by left flank and abdominal pain, with or without unilateral macroscopic or microscopic haematuria. When the venous reflux caused by the LRV entrapment leads to the formation of collaterals this syndrome may be a cause of “pelvic congestion syndrome” characterized by an array of signs and symptoms such as dyspareunia, dysmenorrhoea, lower abdominal pain and pelvic, perineal and lower limb varices [7]. This illness can present quite differently evoking a heightened sense of alert in order to allow an accurate and in time diagnosis. The authors present here a case report of a Posterior Nutcracker Syndrome in a 12-year-old boy with LVR duplication observed in our institution.

## 2. Case Report

The patient is a 12-year-old boy presented to our institution with intermittent gross painless haematuria, which lasted for the 11 months prior to his first under hospital observation. The haemoglobin value on presentation was 8.6 g/dL. He was initially submitted and managed with continuous saline bladder irrigation, not needing blood transfusion or any other treatment.

He denied any other urological signs or symptoms, namely, dysuria or other low urinary tract symptoms (LUTS), flank pain, fever, asthenia, or fatigue. His physical examination was normal. The urine analysis confirmed gross haematuria and no other abnormalities namely, the presence of urinary casts. The immunological markers ANCa and ANA were negative.

**Figure 1:**
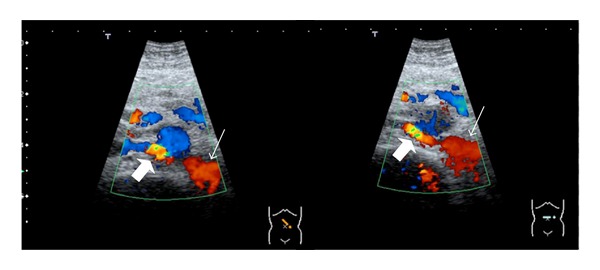
Posterior renal vein branch dilatation (thin arrows) and turbulent flow pattern on the entrapment.

**Figure 2:**
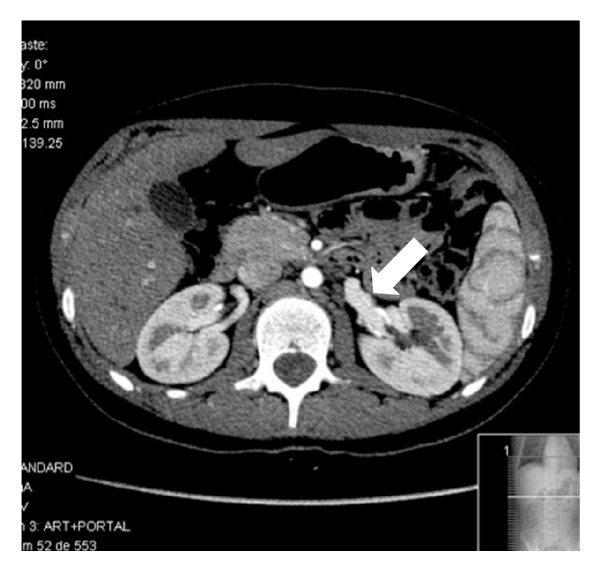
Enlarged left renal vein.

**Figure 3:**
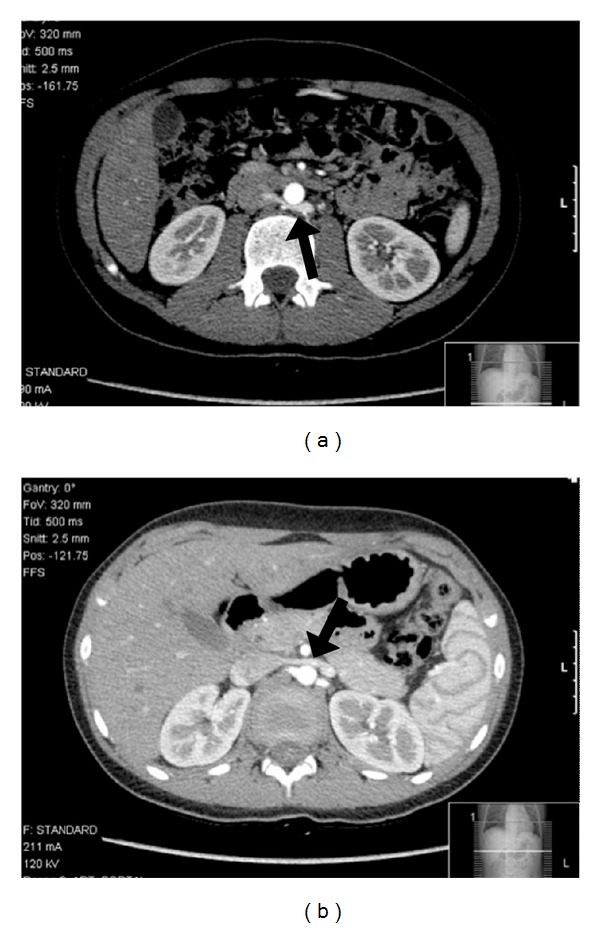
(a) Posterior branch of the left renal vein entrapment; (b) normal anterior left renal Vein.

The ultrasonography showed normal kidney measurements and echogenicity, excluding hydronephrosis, renal masses or stones. The Doppler study revealed turbulent pattern of venous blood flow of the posterior LRV branch behind the aorta (Figure 1). The abdominopelvic computer tomography (apCT) revealed LRV duplication with a dilated retroaortic branch, entrapped between the aorta and the vertebral column, promoting the renal nutcracker syndrome (Figures 2 and 3). The cystoscopy showed normal bladder and urethra mucosa, and a bloody ejaculate only from the left ureter meatus.

Thereafter he was counseled to intense physical exercise restriction, oral iron supplements showing reduction of the haematuria episodes and substantial improvement of anaemia (actual Hg: 10.6 g/dL). The child is currently asymptomatic and proposed to clinical and analytical assessment.

## 3. Discussion

Haematuria is very common and mostly benign in pediatric age [8], but when it subsists longer than 6 months, gross haematuria must be evaluated. The medical history, physical examination, laboratory (complete blood count, electrolytes, creatinine, BUN, and urine culture) [5, 9] and radiographic study (ultrasonography) [10, 11] excluded the most common causes of haematuria such as urinary tract infection, hypercalciuria, intrarenal pathology, and non-glomerular etiology, other etiologies were searched [7, 8, 12]. Observing a bloody ejaculate from the left ureteral meatus, the cystoscopy prompted an evaluation of the left kidney and ureter. The ultrasonography is a good first radiographic study as it is safe, cost effective and screens possible renal and bladder abnormalities [8]. Using Doppler study, it is possible to identify turbulent vessel renal flow [10, 13]. On the other hand CT scan best defines renal masses, stones and may show abnormal renal vessels or abdominal masses causing extrinsic compression [10, 14].

In the nutcracker syndrome, the Doppler ultrasonography usually shows a five time exceeded ratio between the anteroposterior diameters (apDs) and between the peak velocities measured in two different points of the LRV (normally at the level of the renal hilum and the point where the left renal vein is narrowed) [1, 13]. In this case, we could not confirm a peak velocity ratio difference mentioned earlier but could show a high turbulent flow of the posterior LRV branch behind the aorta. The apCT led to this diagnosis by showing a dilated posterior LRV branch being compressed between the aorta and the vertebral column. This single-branch compression is apparently the haematuria promoter by exposing intrarenal veins to higher pressures, which results in the formation of venous collaterals, varices, and subsequent rupture of the septum between the veins and the collecting system.

The renal nutcracker syndrome management evolved in the last four decades [4, 9, 15], and there are several available options, from close expectant surveillance, endoscopic proceedings (e.g., external or internal stenting [16], and haemostatic agents) to more complex open surgical procedures (e.g., LRV transposition, renal autotransplantation of the left kidney [15, 17]). Patients in prepubertal age should be offered less aggressive forms of treatment since the likelihood of spontaneous remission due to normal physical development [6].

The authors present here a rare case of nutcracker syndrome, as the etiology of the haematuria is a single dilated posterior renal vein branch entrapped between the aorta and the vertebral column. This might be the explanation for the intermittent presentation of the gross haematuria. Despite the initial presentation with hospitalization, our patient was managed conservatively with very good results.

## 4. Conclusion

The authors present here a case of a 12-year-old boy with gross asymptomatic haematuria promoted by a posterior Nutcracker Syndrome of the posterior branch of LRV. Our case report shows a rare cause and presentation of a nutcracker syndrome. This is a rare entity, with diverse presentations that affect mostly female young adults and provides a variety of signs and symptoms. This evokes the necessity of a heightened sense of alert when investigating haematuria in children in order to achieve a correct and attempted diagnosis.
